# Differentiating borderline *HER2*-expressing and *HER2*-positive cancers from other subtypes using serum urokinase plasminogen activator

**DOI:** 10.1038/s41416-026-03471-5

**Published:** 2026-05-20

**Authors:** Michael E. J. López Mujica, Suchanat Boonkaew, Nana L. Christensen, Mette Abildgaard Pedersen, Kit Riegels Jørgensen, Mikkel Holm Vendelbo, Elena E. Ferapontova

**Affiliations:** 1 Interdisciplinary Nanoscience Center (iNANO), Faculty of Natural Sciences, Aarhus University, Aarhus C, Denmark; 2 Department of Nuclear Medicine, Aarhus University Hospital, Aarhus N, Denmark; 3 Department of Pathology, Aarhus University Hospital, Aarhus N, Denmark; 4 Department of Biomedicine, Aarhus University, Aarhus C, Denmark

**Keywords:** Diagnostic markers, Cancer screening

## Abstract

**Background:**

*HER2*-positive (*HER2*+) cancers are associated with aggressive tumour development but also high response rates to targeted blockade treatments of the HER-2/*neu* signalling pathway leading to improved clinical outcome for the patient. Current clinical analysis of the *HER2* status primarily relies on solid tumour biopsies low-suitable for continuous real-time monitoring needed for possible adjustment of the treatment, while serum tests targeting blood-circulating HER-2/*neu* fragments often show conflicting tumour-serum relations.

**Methods:**

A cellulase-linked aptamer sandwich assay was used for detection of total urokinase plasminogen activator (uPA) and its different forms in serum of cancer patients and healthy individuals. Serum uPA levels were correlated with solid biopsy results and relevant clinical data extracted from electronic patient records, and FDG-PET/CT scanning.

**Results:**

We show that serum uPA precisely stratifies patients with HER-2/*neu* overexpressing (*HER2*+) and borderline-expressing cancers. Serum levels of total uPA 96.8% accurately informed about HER-2/*neu* tumour status in a cohort of 100 patients, with a *HER2*+/borderline expression cut-off value of 0.973 ng mL^−1^.

**Conclusions:**

The established liquid biopsy test for serum uPA has potential for accurate diagnosis and staging of patients with *HER2*+ and borderline-expressing cancers requiring further confirmatory (or rejection) testing.

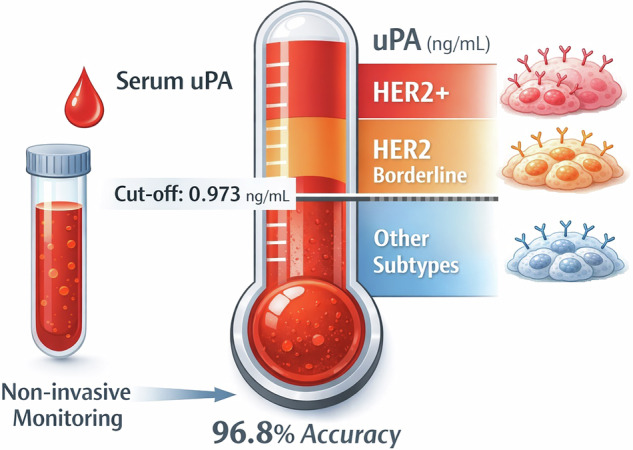

## Background

Despite huge advancements in treatment, cancer remains the second leading cause of death, with almost 10 million fatalities reported in 2020 worldwide [1]. Aggressive forms of breast, oesophageal, lung, liver, and pancreatic cancer have particularly poor prognoses and contribute significantly to the death toll, showing five-year survival rates below 20% [2]. To improve treatment outcomes, more advanced precision medicine approaches are needed, including diagnostic tools for early cancer detection tailored to individual cancers that would ease fast decision-making on which treatment patients will benefit from.

One of the most promising analytical tools, both for early cancer detection and its continuous treatment monitoring, is liquid biopsy [3], defined as a laboratory testing of a sample of body fluids (blood, urine, etc.) for biomolecules/cancer cells released by tumour. By providing non-invasive and easy access to specific tumour biomarkers through a simple blood draw, liquid biopsy is not biased by the selection of the tumour region as solid biopsy. Yet its largest challenge is finding robust liquid-biopsy molecular biomarkers of specific cancers such as tumour-specific proteins currently defined and detected largely by tissue biopsy [4–6].

Human epidermal growth factor receptor-2 (HER-2/*neu*), a glycoprotein complex belonging to the family of receptor tyrosine kinases, refers to such tumour-specific protein biomarkers when overexpressed by tumour cells in a number of aggressive cancers, such as breast, lung, colorectal, bladder, and gastro-oesophageal cancers [7, 8]. *HER2*-positive tumours (showing HER-2/*neu* immunohistochemistry (IHC) score 3+) tend to exhibit aggressive growth and high potential for metastasis associated with poor clinical outcomes [7]. In gastroesophageal cancer patients, *HER2*-positive tumours require targeted blockade treatments of the HER-2/*neu* signalling pathway [9]. The same refers to from 15 to 20% of breast cancers, where *HER2* overexpression correlates with a particularly aggressive tumour development, making HER-2/*neu* targeted therapies important for improving patient prognosis [8, 10]. Concurrently, score 1+/2+ tumours with no *HER2* gene amplification (*HER2*-low breast cancers) also responded well to *HER2*-targeted therapies (the DESTINY-Breast04 trial) [11], which is poorly understood but, in terms of treatment, challenges current classification of *HER2*-associated cancers. Accurate monitoring of the *HER2* tumour status over disease progression is thus key to assessing the response and adjusting the treatment. Yet, current clinical analysis of *HER2* cancer subtypes primarily depends on solid biopsies, providing tissue for detecting expression of the protein itself by IHC, supported by examination of possible amplification of the *HER2* gene through fluorescence in situ hybridisation (FISH) [12, 13]. Both, approved by FDA, are inconvenient to the patient, as multiple biopsies should be taken.

Liquid biopsy analysis of the extracellular domain of HER-2/*neu* (ECD), proteolytically cleaved and released into the bloodstream by tumour cells, was suggested as alternative to solid tumour HER-2/*neu* analysis [12, 13], enhancing diagnostic accuracy [14, 15]. Yet reports on HER-2/*neu* serum diagnostics are inconsistent and show conflicting tumour-serum correlations [13, 15–22]. Other approaches for HER-2/*neu* expression analysis include (1) the assessment of *HER2* copy-number variation (CNV/amplification) in circulating tumour DNA (ctDNA) and (2) the evaluation of *HER2* status in circulating tumour cells (CTCs). The first approach is particularly valuable in metastatic disease [23] and recommended by ESMO in situations where tissue-based testing cannot be performed (tumour tissue is difficult to obtain or insufficient for analysis) or when urgent therapeutic decisions are required [24]. The main limitations include strong dependence on ctDNA fraction, which may result in false negatives in low-shedding tumours, as well as variability related to ploidy, sub-clonality, panel design, and inter-laboratory differences in thresholds and analytical algorithms [24]. The second approach relies on the evaluation of either protein expression or gene amplification in CTC [25]. Yet *HER2* status in CTCs may differ from that of primary tumour tissue, and discordance between tissue and CTC *HER2* status is frequently reported [26, 27]. The clinical utility of this approach remains under investigation and is not yet standardised to the same extent as tissue-based testing [28].

Further evidences occur that urokinase plasminogen activator (uPA), a serine protease implicated in the processes of tumour invasion and metastatic spread [29, 30], is overexpressed in *HER2*-positive tumours [31, 32], with both *HER2* and uPA receptor (uPAR) genes co-amplified most frequently in the same patient’s cancer tissue extracts [32]. uPA is a part of the blood fibrinolytic system inducing peri-cellular proteolysis either by degrading extracellular-matrix (ECM) components or by activating latent proteases or growth factors [33]. Along with its receptor, uPAR, uPA not only triggers a cascade of proteolytic events occurring during tumour invasion and metastatic spread but also plays a larger role in cancer development, from tumorigenesis to metastasis [30, 34]. Hitherto, most studies have focused on evaluation of uPA as a prognostic biomarker of disease-free and overall survival rates [35]. Its expression in tumour cells was correlated with survival rates in node-negative breast cancers [36–38], primary invasive breast cancers [39], primary breast cancers [40], and primary colorectal cancers [41]. Overexpression of uPAR in *HER2*-positive breast tumours was shown to contribute to aggressive metastatic phenotype of cancer with poor clinical outcome [31], and elevated serum uPA in cancers patients, above 2.5 ng mL^−1^, was reported as a cut-off for metastatic vs. non-metastatic breast cancer type and also predicating shorter survival rates [42].

**Scheme 1:**
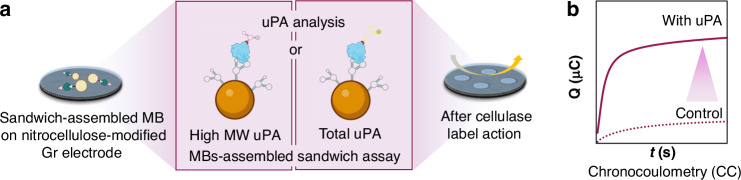
**a** Principle of the uPA detection by the sandwich aptamer1-uPA-aptamer2-cellulase assay on magnetic beads (MBs). The protein binds to the aptamer1-modified MBs, then the cellulase-conjugated reporter aptamer2 binds to uPA, with this completing the sandwich assembly. When applied onto nitrocellulose-modified electrodes, cellulase digests the film, changing its electrical properties, which are (**b**) then read out chronocoulometrically.

Given that uPA is overexpressed in *HER2*-positive breast tumours [31, 32], we hypothesised that serum uPA might be a consistent liquid biopsy biomarker of *HER2*-positive cancers. Here, we assessed serum uPA in 30 healthy volunteers and 100 cancer patients diagnosed with mostly breast and oesophagus cancers of different phenotypes. For serum analysis, we used analytically validated electrochemical cellulase-linked aptamer-sorbent assay (e-ELASA) on magnetic beads [43–45], earlier shown to be an accurate tool for new biomarker’s discovery [45] (Scheme 1). We show that serum uPA correctly informs about HER-2/*neu* overexpressing (*HER2*-positive) and borderline-expressing tumours and appears to be an accurate biomarker of this cancer phenotype.

## Methods

### Materials and reagents

Urokinase (uPA, 411 amino acids; MW: 54 kDa, a two-chain glycoprotein isolated from human urine, specific activity: 187,973 IU mg^−1^) was delivered by ProSpec (Israel) as a sterile filtered lyophilised powder. Aptamers specific for HER-2/*neu* [43, 44] and uPA [46] ordered from Metabion (Germany) were: uPA02 as a capture aptamer for both high molecular weight (HMW uPA) and total uPA (42-mer: 5′-biotin-CAA GCG GGG GTG AGA GAT CTG TCA GTA CGA GCT GGG TTT GCG-3′); uPA08, as a reporter aptamer solely for HMW uPA (41-mer: 5′ amine-C_6_-CAG CGG TAG GGG TTA TAT AGC TGC GCC ATA GGG TAC TCG TG-3′); uPA21, as a reporter aptamer for total uPA (82-mer: 5′ amine-C_6_-AGG TAG AGG AGC AAG CCA TCG GAG GTA CTC ACC GAC GCT GAA CTC CAT AGA ATG TGG TGA TGG ATG CGT GAT CGA ACC TAC C-3′); HER-2/*neu* capture and reporter aptamers (TTT TTT GCA GCG GTG TGG GGG CAG CGG TGT GGG G-3′ modified with either 5′ biotin or C_6_-amine). All stock solutions (proteins, aptamers, and serum samples) were prepared/diluted with a 10 mM phosphate buffer solution containing 150 mM NaCl, pH 7.4 (PBS), at room temperature, rt (20 ± 2 °C), stored at −20 °C until used. Additional details on reagents are in the Supporting Information, **SI**.

### Sandwich aptamer assay

Protocols for electrode handling, streptavidin-modified magnetic beads (MB) functionalization, and cellulase conjugation to reporter aptamers are given in SI. For calibration curves construction, 40 μL of uPA02-modified MBs were mixed with 960 μL of uPA solutions (from 10 aM to 100 pM) either in PBS, pH 7.4, or in 10% serum, and incubated for 30 min at rt with 300 rpm shaking. uPA-spiked serum samples were prepared with human serum from male AB plasma diluted 10 times with PBS. MBs were then washed 3 × 1 mL with 0.1% BSA in PBS, pH 7.4, the BSA solution being decanted. Next, 100 μL of 1 μM cellulase-aptamer bioconjugate (uPA08 or uPA21) were added to MBs and allowed to react for 30 min at rt under 300 rpm shaking, then being washed 3 × 100 μL with 0.1% BSA in PBS. The same protocol was used in HER-2/*neu* aptamer-based assay [44]. After decanting the BSA solution, MBs with fully assembled sandwiches were resuspended in 200 μL of PBS, pH 5, and 5 μL of sandwich suspension were dropped onto the nitrocellulose-modified electrodes and incubated for 20 min at rt. Finally, MBs were water-rinsed from the electrode surface, and the electrodes were chronocoulometrically (CC) tested, at 0.3 V (*t*-interval 0.1 s; *t*-run 10 s). The control (blank) experiments were performed by running the MB-assembly assay without uPA or serum uPA, but only in PBS or serum, with the same electrodes further exposed, after being washed with water, to sandwiches assembled on MBs in sample solutions. The change in the charge ∆*Q* was calculated by subtracting the blank response (*Q*_0_) from the response of uPA-containing samples (*Q*_uPA_). All analytical detections, also with patients’ samples, were performed in triplicates, each time with a new electrode.

### Sample preparation and statistical analysis

Serum samples were prepared as previously described [45]. Briefly, the collected blood samples were centrifuged at 2000 × *g* for 10 min to remove clots, and the resulting supernatant (serum) was then stored at −20 °C until use. All serum samples were diluted 10-fold with PBS and then assayed as described above. Cancer biopsy results and other relevant clinical data (pathology reports and biochemical laboratory results) were extracted from the electronic patient records. Details of the statistical analysis are provided in the **SI**.

## Results

### Analysis of serum HER-2/*neu* and total uPA

**Fig. 1:**
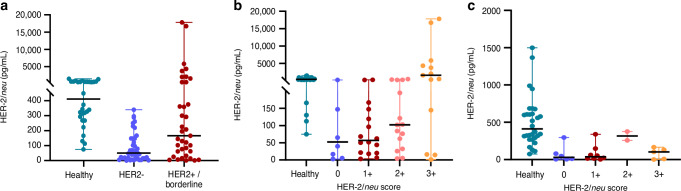
Correlation betwen serum HER-2/*neu* levels and *HER2* tumor status in 77 breast and oesophageal cancer patients. **a** Serum HER-2/*neu* levels in healthy volunteers (*n* = 30), and in patients with *HER2* negative (HER2−) and *HER2*-positive (HER2+) cancers and cancers with borderline expression of HER-2/*neu* (HER2+/borderline) (*n* = 77). Medians: 408, 51, and 165 pg mL^−1^, respectively. **b, c** HER-2/*neu* levels in cancer patients stratified by HER-2/*neu* IHC scores: **b** breast cancer (medians: 368, 52, 52, 102, and 1508 pg mL^−1^, respectively), and **c** oesophagus cancer (medians: 408, 30, 33, 312, and 98 pg mL^−1^, respectively). All serum samples were diluted 10 times before being examined in triplicates. Statistical analysis was carried out using GraphPad Prism 10; black horizontal lines indicate the median.

First, the cellulase-linked e-ELASA assay, previously developed and analytically validated against conventional ELASA for the accurate detection of serum HER-2/*neu* and PSA [43–45, 47], was used to measure serum HER-2/*neu* ECD in patients’ samples (Figs. 1 and  SI). Only a small number of *HER2*-positive cancer cases showed significantly elevated serum HER-2/*neu* levels; in the remaining cases, the values were comparable to those observed in healthy individuals and in cancer patients without HER-2/*neu*–positive subtypes. In most *HER2*-positive cancer cases (score 3+), serum HER-2/*neu* concentrations were below the discussed cut-off values for *HER2*–positive tumours (15 ng mL^−1^ [14] or 37 ng mL^−1^ [13]). Consistent with previous reports [13, 15–22], we observed only a weak correlation between serum HER-2/*neu* levels and tumour status.

Then, cellulase-linked e-ELASA was adapted for analysis of serum uPA by using a couple of uPA02 (capture) and uPA21 (reporter) aptamers specific for total uPA (Scheme 1). These were DNA aptamers, inexpensive compared to the previously used fluorinated RNA aptamer [48] yet similarly stable in serum. Calibration curves were constructed and used for uPA analysis in human samples (Figs. S1A, S1B, S2 and SI). Sensitivity of total uPA detection was (20.28 ± 5.21) µC fM^−1^ in PBS and (19.14 ± 4.50) µC fM^−1^ in serum (relative difference, RD = 6.0%), and the limit of detection (LOD) determined by IUPAC as ‘*the smallest concentration of analyte in the sample that can be reliably distinguished from zero*’ was 1 aM, both in serum and PBS (0.430 aM when calculated as 3×*σ*/*S*, where *σ* is the standard deviation of the blank signal and *S* is the sensitivity of analysis (the slope of the calibration curve).

Serum uPA was then analysed in 30 healthy individuals and 100 cancer patients (64 breast cancer, 30 oesophagus cancer, 5 gastric cancer and 1 cardia cancer) (Tables S1, SI). Of those, 95 patients were diagnosed with different degrees of HER-2/*neu* expression (normal expression correlates with IHC HER-2/*neu* score 0 and 1+, borderline expression—with score 2+, and overexpression—with score 3+ indicating *HER2*-positive cancers [49]), including 22 patients with a borderline expression and 21 patients with an overexpressed HER-2/*neu*. For convenience, this 43 patients’ group is also referred to as ‘allegedly *HER2*-positive’ yet including both scores 3+ and 2+ cases (overexpression and borderline expression of HER-2/*neu)*, of which only former indicated *HER2*-positive cancers. Of 22 score 2+ patients, two had *HER2*-positive diagnosis confirmed after additional testing of the *HER2* gene amplification degree. Further, we provide separate analytical results for the *HER2* borderline expression and overexpression cases. For 5 cancer patients their *HER2* status was not identified and thus considered as *HER2*-negative, yet they were excluded from final analysis (Figs. S3 and SI).

**Fig. 2:**
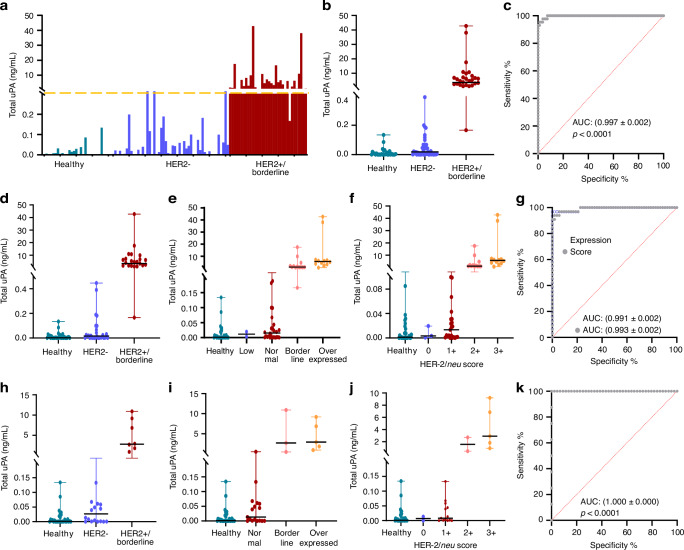
Correlation between serum total uPA levels and *HER2* tumor status in 100 breast and oesophageal cancer patients. **a**, **b** Correlation between the total serum uPA levels and the patients’ *HER2* status based on analysis of serum samples collected from healthy volunteers (*n* = 30), *HER2*-negative patients (low and normally expressed HER-2/*neu*) (*n* = 56) and with different *HER2* types (borderline expressed and overexpressed HER-2/*neu*) (*n* = 32). **c** ROC curves obtained from the total uPA analysis data. **d**–**k** Segmented analysis of total serum uPA in healthy individuals and *HER2*-negative and allegedly *HER2-* positive cancer patients by cancer type: (**d**–**g** panel) breast cancer (*n* = 56) and (**h**–**k** panel) oesophagus cancer (*n* = 26). (**d, e, h, i**) Total serum uPA stratified by the HER-2/*neu* expression levels and (**f, j**) by IHC scores. ROC curves obtained for (**g**) breast and (**k**) oesophagus cancer cases based on HER-2/*neu* expression. All serum samples were diluted 10 times before being examined in triplicates. In plots, black horizontal lines indicate the median, the error bar means the upper and lower values. Statistical analysis was carried out using GraphPad Prism 10.

Serum uPA levels were increased statistically significantly in the allegedly *HER2*-positive 32 patients’ group (a 95% confidence interval), compared to the uPA levels in cancer patients with low and normal expression of HER-2/*neu* and healthy individuals (Fig. 2a, b). The average concentration of total uPA in allegedly *HER2*-positive cases reached (4.468 ± 3.451) ng mL^−1^ (*p* < 0.0001), 200-fold exceeding uPA levels detected in the serum of other cancer patients, (0.023 ± 0.014) ng mL^−1^ (*p* < 0.001), and 444-fold exceeding serum uPA in a healthy cohort, (0.015 ± 0.012) ng mL^−1^ (*p* < 0.001). Only one of 32 analyses was visibly out of range, leading to 96.6% accuracy of the HER-2/*neu* borderline expression and overexpression detection by serum uPA, indicating accurately the extent of HER-2/*neu* expression in both cases.

ROC analysis of the uPA results (Fig. 2c) showed a remarkable assay performance, with close to excellent AUC of (0.997 ± 0.002) (*p* < 0.0001). The cut-off value derived was 0.973 ng mL^−1^. The ROC curve’s proximity to the upper-left corner of the ROC space reflected the optimal balance of sensitivity and specificity, confirming accuracy of the HER-2/*neu* borderline expression and overexpression discrimination from other cases by serum uPA analysis.

It is important to note that in the 43 patients’ group (with borderline expressed and overexpressed HER-2/*neu*), 11 patients were treated and supposedly cured to the moment of their serum samples collection synchronised with FDG-PET/CT scanning that showed no tumour present. Their serum uPA results were excluded from the data analysis shown in Fig. 2, their analysis results being provided in Figs. S3 and SI. Nine of these patients had serum uPA levels above the cut-off value. For *HER2*-negative cancers, eight cured patients’ data were similarly excluded.

Total uPA results were stratified by cancer type: breast cancer (*n* = 56; Fig. 2d–g, with eight cured/unidentified cases excluded and represented in Fig. S3) and oesophagus cancer (*n* = 26; Fig. 2h–k, with four cured/unidentified cases transferred to Fig. S3). Six cases of gastric and cardia cancer were excluded from comparative analysis of organ specific cancers as they were *HER2*-negative; their serum total uPA was below 0.92 ng mL^−1^ (Figs. S4 and SI). Both allegedly *HER2*-positive specific cancers showed notably high serum uPA levels, on average, (5.751 ± 4.473) ng mL^−1^ (*p* < 0.0001) in breast cancer and (3.366 ± 2.175) ng mL^−1^ (*p* < 0.0001) in oesophagus cancer, exceeding 293-fold and 445-fold average uPA concentrations in the serum of *HER2*-negative breast and oesophagus cancer patients (Fig. 2d, h). Thus, elevated serum uPA correlated with a borderline expression and overexpression of HER-2/*neu* (Fig. 2e, i). In breast cancer, the borderline expression of HER-2/*neu* correlated with the average (3.829 ± 2.194) ng mL^−1^ (*p* < 0.0001) serum uPA, increasing to (11.503 ± 9.297) ng mL^−1^ (*p* < 0.0001) in the case of HER-2/*neu* overexpression. For comparison, it was just (0.011 ± 0.009) ng mL^−1^ (*p* < 0.001) in serum of patients with normal HER-2/*neu* expression. In oesophagus cancer, the borderline expression correlated with an average (1.575 ± 0.986) ng mL^−1^ serum uPA (*p* < 0.001), and overexpression - with (3.301 ± 1.991) ng mL^−1^ uPA (*p* < 0.0001), with normal HER-2/*neu* expression resulting in the meaningly lower serum uPA of (0.010 ± 0.009) ng mL^−1^ (*p* < 0.001). Similarly, HER-2/*neu* scores 2+ and 3+, reflecting a high-density HER-2/*neu* population on the tumour cell surface, can be linked to higher serum uPA concentrations compared with those observed in cancer patients with *HER2*-negative tumours (scores 0 and 1+) (Fig. 2f, j).

ROC curve analysis of serum total uPA data gave quite close cut-off values for *HER2*-positive cancers: 0.877 ng mL⁻¹ uPA for breast cancer (AUC: 0.991 ± 0.002 (*p* < 0.0001)) and 0.853 ng mL⁻¹ uPA for oesophageal cancer (AUC: 1.000 ± 0.000) (*p* < 0.0001) (Fig. 2g, k). Considering other parameters, no correlation was found between serum uPA and patients’ sex, weight/height, age, oestrogen receptor status, tumour burden and metastatic state (Figs. S5–7 and SI).

### Analysis of specific blood-circulating forms of uPA

**Fig. 3:**
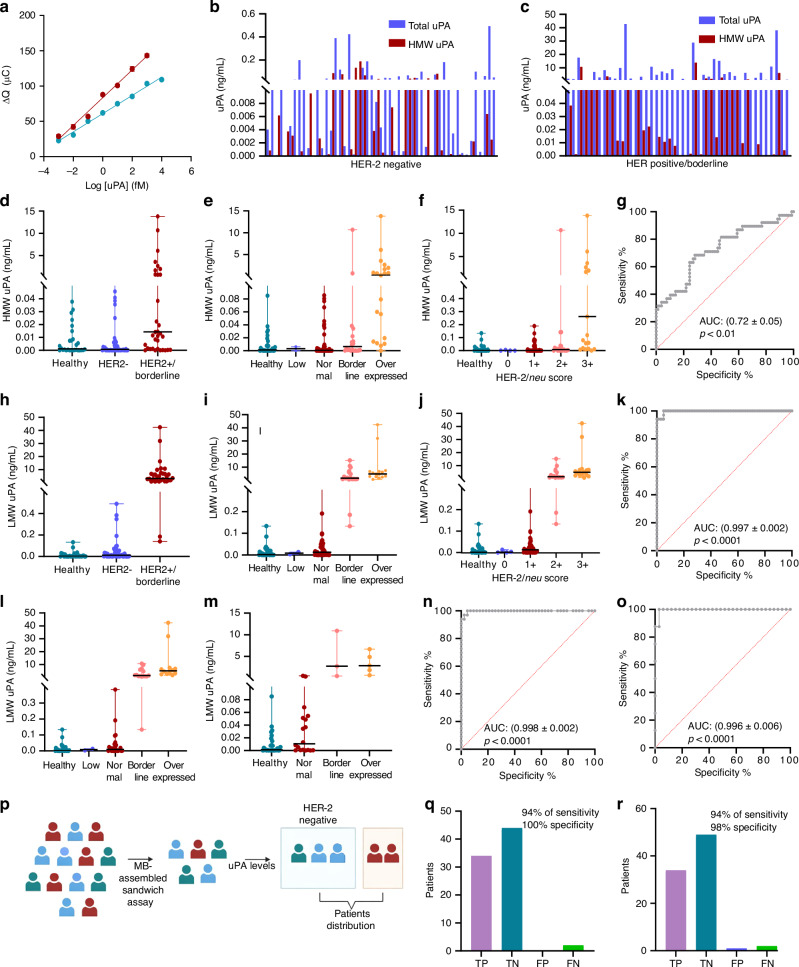
Correlation between serum HMW and LMW uPA levels and *HER2* tumor status in 100 breast and oesophageal cancer patients. **a** The semi-logarithmic dependences of e-ELASA responses on the total uPA (cyan) and HMW uPA (wine) concentrations in uPA-spiked 10% human (error bars are smaller than some concentration dots). **b**,** c** Correlation between the serum total uPA (blue) and HMW uPA (red) levels and the patients’ *HER2* status based on analysis of serum samples from cancer patients with not overexpressed HER-2/*neu* (*n* = 52) and with allegedly *HER2* positive types (*n* = 32). Healthy volunteers’ data (*n* = 30) are in Figs. S9 and SI. **d**–**f** Serum HMW uPA and (**h**–**j**) LMW uPA levels stratified by (**d**,** h**) patients’ cancer status, (**e**,** i**) HER-2/*neu* expression levels, and (**f**,** J**) ICH scores. **g**,** k** ROC curves obtained for (**g**) HMW uPA and (**k**) LMW uPA analysis of tumour’s *HER2* status based on HER-2/*neu* expression (**l**,** m**) Serum LMW uPA levels stratified by HER-2/*neu* expression levels for (**l**) breast cancer and (**m**) oesophageal cancer; and (**n**,** o**) corresponding ROC curves for (**n**) breast cancer and (**o**) oesophageal cancer based on HER-2/*neu* expression. **p** Scheme of patients´ liquid biopsy analysis. The value of serum uPA analysis for borderline expression and overexpression of HER-2/*neu*: true positives (TP), true negatives (TN), false positives (FP) and false negatives (FN) of the allegedly *HER2*-positive group obtained by (**q**) detecting the total uPA and (**r**) detecting LMW uPA. The sensitivity calculated as TN/(TN + FP) and the selectivity calculated as TP/(TP + FN). The rest notations/conditions are as in Fig. 1 (samples preparation and statistics).

In body, uPA exists in several isoforms: its originally expressed pro-uPA zymogen form, high molecular weight uPA (HMW uPA), and low molecular weight uPA (LMW uPA) [30]. In Section ‘Analysis of serum HER-2/neu and total uPA’, we used aptamers recognising the sum of all isoforms referred to as total uPA. Yet, in the blood, uPA circulates in two main catalytically active isoforms: HMW uPA and LMW uPA, both may be involved in tumour progression. To determine which form most accurately reflects and/or contributes to *HER2*-associated uPA response, we adapted e-ELASA to selectively detect HMW uPA, by using as a reporter uPA08 aptamer specific solely for HMW uPA [46]. As in total uPA analysis, calibration curves first were constructed for HMW uPA analysis in human serum samples (Figs. 3a, S8 and SI). Sensitivity of HMW uPA detection in serum, 12.912 µC fM^−1^, was close to that in PBS, and the LOD was 1 aM (by IUPAC definition) and 0.430 aM (as previously specified). The calibration curves linear equations and sensitivities of analysis were:

Total uPA analysis in PBS: *y* = 20.28*x* + 83.87, *R*² = 0.99 (the sensitivity: 20.28 ± 5.21 μC fM^−1^), and in 10% serum: *y* = 19.14*x* + 82.93, *R*² = 0.99 (the sensitivity: 19.14 ± 4.5 μC fM^−1^).

HMW uPA analysis in PBS: *y* = 13.40*x* + 63.96, *R*² = 0.99 (the sensitivity: 13.40 ± 3.23 μC fM^−1^), and 10% serum: *y* = 12.91*x* + 60.93, *R*² = 0.99 (the sensitivity: 12.91 ± 3.03 μC fM^−1^).

Both serum HMW uPA and LMW uPA levels were analysed in serum samples of patients with tumours showing borderline expression of HER-2/*neu* and in *HER2*-positive tumours, by using the total uPA and HMW uPA specific e-ELASAs. LMW uPA was calculated by subtracting the detected HMW uPA concentration from the total uPA concentration. This dual assessment provided deeper insights into serum uPA profiles in patients with different HER-2/*neu* tumour statuses. We found that serum HMW uPA did not show clear variation patterns as total uPA did, indicating that serum HMW uPA contributed weakly to the accuracy of *HER2*-associated cancer status determination (Fig. 3b–g). Although median HMW uPA levels were higher in the groups with overexpressed (0.801 ng mL^−1^; IQR: 0.001 ng mL^−1^–1.617 ng mL^−1^; *p* < 0.09) and borderline expressed *HER2* (0.013 ng mL^−1^; IQR: 0.002 ng mL^−1^–0.038 ng mL^−1^; *p* < 0.48) than in *HER2*-negative (0.023 ng mL^−1^; IQR: 0.0002 ng mL^−1^–0.0350 ng mL^−1^; *p* < 0.31) and healthy individuals (0.011 ng m^−^^1^; IQR: 0.001 ng mL^−1^–0.018 ng mL^−1^; *p* < 0.5) groups, the differences were not statistically significant. The standard deviations were too large, and HMW uPA values overlapped largely across all groups (Fig. 3d–f). A very weak (if any) correlation was found between serum HMW uPA and tumour status, HER-2/*neu* expression and scores. The ROC analysis yielded an AUC of 0.72 ± 0.05 (*p* < 0.01), indicating poor ability of HMW uPA to stratify *HER2* status of tumours.

In contrast, serum LMW uPA correlated remarkably well both with HER-2/*neu* expression levels and scores (Fig. 3h–j). Notably, LMW uPA constituted the major fraction of total uPA, and concentrations of both forms showed strong correlations across all studied groups, in contrast to HMW uPA (Figs. S10 and SI). Serum LMW uPA levels’ average was statistically significantly higher in patients with HER-2/*neu* overexpression (6.697 ± 4.479) ng mL^−1^ (*p* < 0.0001) and with borderline expression (3.201 ± 2.311) ng mL^−1^ (*p* < 0.0001) than in *HER2*-negative (0.042 ± 0.030) ng mL^−1^ (*p* < 0.001) and healthy individuals (0.013 ± 0.010) ng mL^−1^ (*p* < 0.001) groups. ROC curve analysis gave an AUC of 0.997 ± 0.002 (*p* < 0.0001) supporting the diagnostic utility of LMW uPA as a biomarker for HER-2/*neu* borderline-expressed and overexpressed cancers, with a cut-off value of 0.943 ng mL^−1^.

The accuracy of HER-2/*neu* borderline-expressed and overexpressed cancer detection by serum LMW uPA analysis remained similarly high across different cancer types (Fig. 3l, m). In both breast and oesophageal cancer cohorts, the average LMW uPA concentrations were markedly elevated in HER-2/*neu*-overexpressed ((6.436 ± 4.166) ng mL^−1^; *p* < 0.0001 for breast cancer, and (3.596 ± 2.431) ng mL^−1^; *p* < 0.0001 for oesophageal cancer) and borderline-expressed (3.366 ± 1.992) ng mL^−1^; *p* < 0.0001 for breast cancer, and (1.575 ± 1.021) ng mL^−1^; *p* < 0.0001 for oesophageal cancer) cases compared to *HER2*-negative cases ((0.053 ± 0.018) ng mL^−1^; *p* < 0.0001, and (0.035 ± 0.010) ng mL^−1^; *p* < 0.0001, for breast and oesophageal cancer, respectively). These differences were statistically significant and enabled clear distinctions between *HER2*-negative and allegedly *HER2*-positive cancers, with no overlap between groups. ROC curve analysis yielded exceptional AUCs of 0.998 ± 0.002 (*p* < 0.0001) for breast cancer (Fig. 3n), and 0.996 ± 0.006 (*p* < 0.0001), for oesophageal cancer (Fig. 3o), and cut-offs for allegedly *HER2*-positive cancers of 0.910 ng mL^−1^ LMW uPA for the overall cancer dataset, 0.853 ng mL^−1^ for breast cancer, and 0.836 ng mL^−1^ for oesophageal cancer.

Thus, serum total uPA correctly identified 31 out of 32 patients with HER-2/*neu*-overexpressing and borderline-expressing tumours as true positives, with one case classified as false positive, resulting in the cancer stratification accuracy of 96.8% (Fig. 3p, q). Serum LMW uPA allowed 94.1% accurate discrimination of *HER2*-positive tumours and tumours with borderline-expressed HER-2/*neu* (Fig. 3p, r).

## Discussion

Our data show that serum total uPA informs 96.6% accurately about the *HER2* tumour status (HER-2/*neu* borderline expression and overexpression) and thus enables these cancer subtypes stratification. However, serum uPA levels in patients with cancers characterised by HER-2/*neu* borderline expression (including two confirmed *HER2*-positive cases) and overexpression (*HER2*-positive cancers) showed similar patterns and cannot be distinguished. The total uPA concentration above 0.974 ng mL^−1^ (the cut-off value) informed correctly about the HER-2/*neu* expression status of tumours (borderline-expressed or overexpressed) in 32 patients, with one patient giving a false negative result. Serum uPA informed about HER-2/*neu* expression levels independently of such parameters as patients’ sex, weight/height, age, oestrogen receptor status, tumour size, and metastatic conditions. An important observation is that six of the eight diagnosed *HER*2–positive cancer patients with no PET-detectable tumours at the time of serum collection and analysis still had high serum uPA levels (Figs. S3 and SI). As we lack sufficient data to determine whether these cases represent true remission or relapse, we simply acknowledge this observation. We can speculate, however, that uPA overexpression may occur independently of anti-*HER2* treatment, and that elevated serum uPA in patients considered to be in remission might have prognostic value. These hypotheses need to be validated in a larger cohort of treated patients with longitudinal serum analyses; however, the inability to perform such studies within the scope of the present work constitutes its main limitation. Another limitation is the lack of data regarding the effects of inflammatory and coagulation-related conditions on serum uPA levels in HER-2/*neu* overexpressing and borderline-expressing cancers, which would help account for potential inflammatory confounders. Our data demonstrate a statistically significant correlation between elevated serum uPA and HER-2/*neu* expression levels. However, because uPA plays a direct role in the fibrinolytic system, and since systemic inflammation, thrombosis, infection, or recent surgery may influence serum uPA concentrations, additional studies are warranted to further substantiate the *HER2*-specific diagnostic utility of uPA.

Therewith, analysis of different forms of uPA, HMW uPA and LMW uPA, shows that LMW uPA and not HMW uPA correctly informs about the *HER*2 status of tumours. When contribution of serum HMW uPA to total uPA is disregarded, serum LMW uPA informs 96% specifically about *HER2* expression status in all types of cancers studied (breast, oesophagus, gastric, and cardia cancers, Fig. 3h–k), with a *HER2* borderline expression and *HER2*-positive cancer cut-off of 0.910 ng mL^-1^. Both breast cancer and oesophagus cancer cut-offs showed close values.

Another important observation is that serum LMW uPA and total uPA levels in healthy individuals and in *HER2*-negative breast, oesophagus, gastric and cardia carcinomas and adenocarcinomas are not so different, though the median value was slightly higher in cancer patients with normally expressed HER-2/*neu* and HER-2/*neu* score 1+ (Fig. 3j). Thus, serum LMW uPA did not inform generally about cancer. Our results underscore the strong discriminatory capacity of serum uPA to differentiate specifically between *HER2*-negative (score 0 and 1+) and HER-2/*neu* overexpressing and borderline-expressing cancers (scores 2+ and 3+). However, they also raise questions about the underlying pathophysiological processes.

Normally, uPA converts plasminogen to plasmin, a broad-spectrum serine protease that promotes ECM degradation both directly and indirectly, via activation of matrix metalloproteinases, contributing to fibrinolysis by degrading fibrin, the main protein component of blood clots, and some other ECM components [30, 50]. In a healthy organism, plasmin is active in a variety of tissue-remodelling processes helping preventing blood clots, thrombosis, cardiovascular diseases, combatting inflammation and ensure proper wound healing [51]. Yet, in cancer, activation of plasminogen by uPA not only assists proteolytic degradation of ECM, but also causes several pathological processes contributing to tumorigenesis, tumour invasion and metastasis [50]. The reasons for emerging pathophysiological routes are not completely clear, as well as the role played by different catalytically active forms of uPA.

**Fig. 4:**
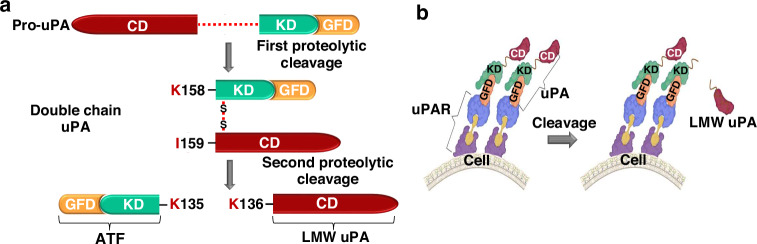
Schematic representation of the uPA activation and cleavage. **a** Schematic representation of the uPA activation and proteolytic cleavage. The single-chain precursor form, pro-uPA, contains 3 structural domains: the growth factor domain (GFD), kringle domain (KD), and catalytic domain (CD). Activation of pro-uPA occurs via cleavage at the Lys158–Ile159 peptide bond, producing the two-chain uPA held together by a disulphide bridge (HMW uPA, ca. 54 kDa). 2nd proteolytic cleavage between Lys135 and Lys136 yields two fragments: the amino-terminal fragment (ATF), including GFD-KD, and the catalytic LMW form (LMW uPA, ca. 33 kDa), comprising the serine protease domain. Adapted from [30]. **b** uPA binding to uPAR dimers on the cell surface facilitates 2nd proteolytic cleavage leading to LMW uPA release into the bloodstream.

uPA is secreted as a single-chain catalytically inactive zymogen form, referred to as pro-uPA, comprising three distinct domains: the growth factor domain (GFD), the kringle domain (KD), and the catalytic serine protease domain (Fig. 4a) [30, 50]. Pro-uPA is then proteolytic cleaved and converted, reportedly by other proteases such as plasmin, trypsin, kallikrein, cathepsin B, human T cell associated serine proteinase-1, and thermolysin [30], into catalytically active double-chain HMW uPA, in which the GFD-KD region (an A chain, involved in binding to receptors and substrates) remains connected to the catalytic domain (a B chain responsible for its enzymatic activity) via a disulphide bond (Fig. 4a).

Further proteolytic cleavage of HMW uPA produces the amino terminal fragment, ATF, comprising the GFD-KD region, capable of binding to uPAR, and a soluble LMW form, which is the catalytic domain and the most part of the structurally disordered 27-residue linker, via which it was tethered to ATF (Fig. 4). This cleavage happens extracellularly, supposedly after uPA binds to its receptor uPAR on the cell surface; yet the exact mechanism remains unclear [52]. Binding of both pro-uPA and HMW uPA to the cell-surface anchored uPAR activates surface conversion of plasminogen to plasmin and may indeed trigger the pathophysiological pathways of tumour development [30].

There are two questions concerning the relation between serum uPA and *HER2*-positive cancers:

1. Why is uPA co-overexpressed with HER-2/*neu* [32, 53]?
2. Why does LMW uPA and not HMW uPA consistently elevate in the blood of patients with tumours overexpressing and borderline-expressing HER-2/*neu*?

Regarding question (1), it was suggested that in *HER2-*positive cancers activation of *HER2* induced strong signalling via the protein kinase Cα and steroid receptor co-activator, both being critical components of the uPA/uPAR-mediated cancer cell invasion, or nuclear factor-kappaB, capable of mediating HER-2/*neu* and uPAR expression in cancer stem cells [31]. This could lead to activation of ETS and Kruppel-like family of transcription factors, whose binding to the promoter regions of *HER2* or *PLAUR* (gene symbol for uPAR) gene then leads to their co-amplification and consequent overexpression of HER-2/*neu* and both uPAR and uPA, since the latter two strongly correlate [31].

Regarding question (2), LMW uPA cannot bind to uPAR and remains soluble, while still retaining full catalytic activity. When the uPA/uPAR system is overexpressed, a proportionally greater fraction of LMW uPA in total uPA can freely circulate in the blood. Pro-uPA and HMW uPA are predominantly cell-bound forms and should therefore constitute a proportionally smaller fraction of serum uPA. However, why is LMW uPA (and not HMW uPA) elevated? We suggest it is closely connected with the way plasmin-uPA system is activated on the cell surface. By cleaving and activating each other, uPA and plasmin establish a positive feedback loop. It is debated how the whole catalytic plasmin-uPA activation circle starts. Recent in vitro studies show that HMW uPA can both activate pro-uPA and undergo autocatalytic self-cleavage releasing LMW uPA, an activity that plasmin cannot perform [52]. Then, the autocatalytic cleavage of HMW uPA may be the most plausible mechanism for the LMW uPA shedding into the bloodstream, since:

(i) Several studies report on uPAR dimerisation on the cell surface and uPA binding to uPAR dimers [52, 54, 55]. Overexpression of uPA/uPAR in *HER2*-positive and HER-2/*neu* borderline-expressing tumours then results in high-density uPA/uPAR dimer complexes on the cell surface.
(ii) Binding of uPA to the dimers brings two HMW uPA (or pro-uPA) molecules capable of autoactivation, in the absence of plasmin [52], in close proximity, which promotes autocatalytic proteolytic cleavage of uPA releasing LMW uPA (Fig. 4b). Then, uPA/uPAR overexpression in tumours overexpressing and borderline-expressing HER-2/*neu* can indeed lead to higher levels of LMW uPA in the blood of such cancer patients.

We compared our findings with previously published data. Most studies focus on uPA analysis in tumours and link elevated tumour uPA with higher disease risk and poorer prognosis [29, 30, 36, 39, 56–62]. Serum uPA state and status may inherently differ from those in tissues. However, serum studies are limited and primarily focused on the prognostic significance of serum uPA, examining connections between total uPA and cancer survival outcomes [42, 63–66].

In head and neck squamous cell carcinomas, the median concentration of serum pro-uPA and HMW uPA was 0.48 ng mL^−^^1^ (range: 0.24–1.92 ng mL^−1^, *p*-value ‘*not specified*’), with no significant difference between healthy volunteers (*n* = 28) and cancer patients (*n* = 40) [63]. The data exhibited high variability, interfering with meaningful statistical analysis. Another study linked elevated serum uPAR, rather than total uPA (median: 0.69 ng mL^−1^; range: 0-4.76 ng mL^−1^), to an increased risk of soft-tissue sarcoma-related mortality, with no correlation between serum uPA and clinicopathological parameters (*n* = 79) [64]. Patients with pancreatic ductal adenocarcinoma showed a mean serum uPA level of 3.23 ± 1.84 ng mL^−^^1^ (range: 1.24–7.6 ng mL^−1^; median: 2.75 ng mL^−1^), compared to 2.18 ± 1.45 ng mL^−1^ in chronic pancreatitis patients (range: 0.88–5.4 ng mL^−1^; median: 1.51 ng mL^−1^) and 1.01 ± 0.32 ng mL^−1^ in the control group (range: 0.2–1.66 ng mL^−1^; median: 1.05 ng mL^−1^). Although the differences among the three groups were statistically significant (*p* < 0.01), substantial overlap of the results and high variability in serum uPA analysis limited the diagnostic accuracy [66]. Correlations between elevated serum uPA (>2 ng mL^−^^1^) and reduced patients’ survival (181 ± 155 days vs. 335 ± 314 days for < 2 ng mL^−1^ uPA; *r* = −0.391; *p* < 0.05) also exhibited high variability [66]. A serum uPA cut-off of 1 ng mL^−1^ predicted hepatocellular carcinoma-related death with a 41% sensitivity and 77.5% specificity (*n* = 287, *p* < 0.002) [65]. The median serum uPA concentration was reported as 0.7 ng mL^−1^ (range 0.2–14.7 ng mL^−1^), or as a mean: 1.0 ± 1.36 ng mL^−1^. A study of metastatic breast cancer patients (*n* = 252) associated serum uPA (cut-off 2.52 ng mL^−1^) with shorter overall and progression-free survival (*p* < 0.001) and the presence of metastases [42]. The high variability of reported results may be attributed to uncertainties regarding the quality of the ELISA kits used for analysis [42, 64–66], currently unavailable on the market. Results were often compromised by inconsistent methodological details provided. More accurate analytical tools might yield more accurate data linking serum uPA and cancer statuses.

Nevertheless, our findings, together with those of others, indicate the existence of two distinct serum uPA patterns in cancer. Elevated uPA levels can be linked to either (1) poor prognosis and metastatic disease or (2) *HER2*-associated cancers that may be also extended to other cancer subtypes driven by similar pathophysiological mechanisms. LMW uPA, dominating in the blood of cancer patients, may play a role in cancer progression due to the loss of regulatory domains and persistence of unregulated protease activity. At its elevated levels, LMW uPA may lead to diffuse-enhanced ECM degradation, promoting invasion and metastasis, and contribute to more aggressive tumour behaviour through uncontrolled ECM remodelling and activation of other proteases. However, our currently limited data (e.g. Figs S7 and SI) do not support a specific role for uPA in metastatic cancers and instead suggest its immediate involvement in *HER2*-associated cancers.

To conclude, we have shown that liquid biopsy testing of serum uPA enables stratification of *HER2*-positive and HER-2/*neu* borderline-expressing cancers. In 100 patients’ trial, with 30 healthy controls, serum total uPA exceeding 0.973 ng mL^−1^ 96.8% accurately informed about HER-2/*neu* expression levels in the HER-2/*neu* score 2+/3+ patients’ group. This novel serum uPA-based classification of HER-2/*neu* overexpressing and borderline-expressing cancers may facilitate the identification of patients most likely to benefit from treatment; however, it requires further validation, including longitudinal outcome studies and assessment of responses to *HER2*-targeted therapies.

## Supplementary information

Supporting Information to the paper

Supporting Information: Table S1

## Data Availability

Data supporting the results reported in the article can be found in the SI files. Any additional data can be provided by request.
