# Supplementary material for: Differentiating borderline HER2-expressing and HER2-positive cancers from other subtypes using serum urokinase plasminogen activator
Source: Br J Cancer. 2026 May 20;135(4):649–58. doi: 10.1038/s41416-026-03471-5 (PMC13427726; doi:10.1038/s41416-026-03471-5)
Supplement: Supplementary file 1 — Supporting Information to the paper [file 41416_2026_3471_MOESM1_ESM.pdf]

Supplementary Information

Differentiating Borderline *HER2*-Expressing and *HER2*-Positive Cancers from Other Subtypes Using Serum Urokinase Plasminogen Activator

Michael E. J. López Mujica[a#], Suchanat Boonkaew[a#], Nana Louise Christensen[b], Mette Abildgaard Pedersen[d], Kit Riegels Jørgensen[b], Mikkel H. Vendelbo[b,c], Elena E. Ferapontova[a*]

[a] *Interdisciplinary Nanoscience Center (iNANO), Faculty of Natural Sciences, Aarhus University, Gustav Wieds Vej 14, 8000 Aarhus C, Denmark*
[b] *Department of Nuclear Medicine, Aarhus University Hospital, Palle Juul-Jensens Boulevard 99, DK-8200 Aarhus N, Denmark*
[c] *Department of Biomedicine, Aarhus University, Høegh-Guldbergs Gade 10, 8000 Aarhus C, Denmark*
[d] *Department of Pathology, Aarhus University Hospital, Palle Juul-Jensens Boulevard 99, DK-8200 Aarhus N, Denmark*

[#] - equal contribution

[*]Corresponding author: E-mail: elena.ferapontova@inano.au.dk (E.E. Ferapontova)

Supplementary details of Materials and Methods:

Materials and reagents

All chemical reagents were of analytical grade and used directly as received. Streptavidin-coated Dynabeads™ MyOne T1 magnetic beads (MBs, Invitrogen, 1 μM in diameter) and human thrombin (Invitrogen) were purchased from Thermo Fisher Scientific (Denmark). Recombinant human HER-2/*neu* ECD (636 amino acids, MW: 110 kDa) was purchased from SinoBiological Inc. (Beijing, P.R. China). Human and Bovine Serum Albumins (HSA and BSA), *N*-hydroxysuccinimide (NHS), N-(3-dimethylaminopropyl)-N'-ethylcarbodiimide-hydrochloride (EDC), *Aspergillus niger* cellulase, 4% collodion solution, 99.9% absolute ethanol, components of buffer solutions, and human serum from male AB plasma (sterile-filtered, batch SLCQ2792; stored at -20 º C) were ordered from Sigma-Aldrich (Denmark). All aqueous solutions were prepared using ultrapure water (18 MΩ cm^{-1}) from a Millipore Milli-Q Reference A+ system (Merck, Denmark).

Graphite electrode modification

Graphite (Gr) rods for working electrodes (WE) were ordered from Electron Microscopy Sciences, Hatfield, USA (cat. number 70200, 3 mm diameter). The Gr rods were cut to a needed length and fitted into the home-made Teflon holders. The electrodes were then polished with emery paper (SIC paper #1000, HV 30−800, Struers, Denmark) for 30 s to get a smooth surface, and then on A4 paper (Papyrus AB, 80 gm^{-2}, Mölndal, Sweden) for another 30 s to get a mirror-like surface. After that, 10 μL of 0.5% nitrocellulose solution produced by diluting the collodion solution with ethanol were placed on the Gr surface and left to dry at rt for at least 15 min. The modified electrodes were then used in the assay.

Modification of the magnetic beads (MBs)

Streptavidin-coated MBs were modified with a biotinylated uPA02 aptamer specific for uPA (alternatively, in the HER-2/*neu* assay, with a biotinylated dimeric aptamer specific for HER-

2/*neu*). For this, 200 µL of a 10 mg mL^{-1} MBs solution were first washed 3×200 µL of 0.1% BSA in PBS, using a DynaMag 2 stand (Fisher Scientific) for magnetic separation. Following separation, the 0.1% BSA solution was decanted. 200 µL of an 8 µM solution of biotinylated uPA02 were then added to washed MBs and incubated for 1 h at rt with shaking at 300 rpm. Next, uPA02-modified MBs were washed (3 × 200 µL) with 0.1% BSA in PBS, decanted, and resuspended in 200 µL of 0.1% BSA in PBS. Suspensions of modified MBs were stored at 4°C without any decrease in their binding properties for at least 2 months.

Aptamer conjugation with cellulase

Cellulase label was conjugated to the NH_2-group of the reporter aptamers specific for high MW uPA (uPA08) and total uPA (uPA21), and HER-2/*neu*, following the previously reported protocol [1]. Briefly, 13.5 µL of 100 µM aptamer solutions in PBS were mixed with 5.15 µL of 48.7 mM NHS/H_2O, 10 µL of 500 mM EDC/H_2O, 2.52 µL of 515 mg mL^{-1} cellulase (19.8 mM, may be 5 min sonicated for better dissolution) in 0.1 M phosphate buffer solution/150 mM NaCl, pH 5 (PBS, pH 5, a cellulase activity optimum [2]), and 18.8 µL of the same buffer solution, to the final volume of 50 µL. The mixture was incubated for 2 h at rt while shaking at 300 rpm, then stored at -20°C.

Electrochemical detection of uPA (the electrochemical set-up used)

Electrochemical measurements were carried out using a EmStat Blue potentiostat (PalmSens BV, Netherlands), controlled by PStrace software v. 5.10, in a three-electrode cell containing 40 mL of PBS, pH 7.4. All experiments were performed at rt in a Faraday cage. Gr electrodes served as a WE, a Pt wire - as a counter electrode, and Ag/AgCl (3 M KCl) - as a reference electrode (RE).

Electrochemical detection of HER-2/*neu*-ECD

Calibration curves for HER-2/*neu* assaying were, as measured in PBS: $y = 20.34 \log_{[HER\text{-}2/neu]} (fM) + 25.66$, $R^2 = 0.991$ (sensitivity 20.34 ± 3.09 μC fM^{-1}), and in 10% serum: $y = 16.18 \log_{[HER\text{-}2/neu]} (fM) + 23.20$, $R^2 = 0.997$ (sensitivity 16.18 ± 5.09 μC fM^{-1}).

Statistical analysis of the data

Unless specified otherwise, data were analyzed using GraphPad Prism 10 (GraphPad Software) and Sigma Plot v.13. Differences between groups were assessed using a two-tailed unpaired Student's t-test, and statistical significance was defined as $p < 0.001$. For such data homogeneity of variance was assumed. Continuous variables are presented as average ± standard deviation (SD) or median, with interquartile range (IQR). Receiver operating characteristic (ROC) curves were constructed to assess the diagnostic performance of the biomarkers by plotting sensitivity (true positive rate) on the y-axis against 1 − specificity (false positive rate) on the x-axis across a range of threshold values. The area under the curve (AUC) was used as a quantitative measure of overall diagnostic accuracy, with higher AUC values indicating greater discriminatory ability. Optimal cut-off values for biomarker expression were determined using the Youden index (YI = sensitivity + specificity − 1), which identifies the threshold that maximizes the combined sensitivity and specificity. For group comparisons, an unpaired two-tailed t-test was applied, and a p-value < 0.05 was considered statistically significant. In all scatter plots, the central line represents the median. Box-and-whisker plots (in Sigma Plot) were also used to graphically represent the distribution of continuous variables. Each box indicates each IQR, which comprises the 25th (Q1) to the 75th percentile (Q3) of the data. The horizontal line inside the box represents the median (Q2). The whiskers extend to the smallest and largest values within 1.5 times the IQR from the lower and upper quartiles, respectively.

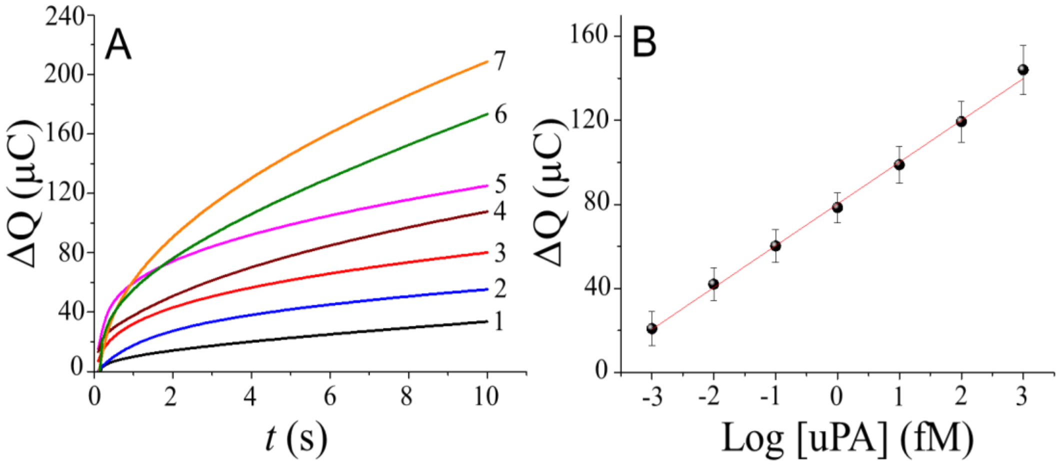

Figure S1. (**A**) Representative CC responses to different concentrations of total uPA ranging from 1.0 aM to 1.0 pM in PBS: (1) 1.0 aM (black), (2) 10 aM (blue), (3) 0.1 fM (red), (4) 1.0 fM (wine), (5) 10 fM (magenta), (6) 100 fM (olive green), (7) 1.0 pM (orange). (**B**) Calibration plot extracted from data shown in (**A**). Responses were recorded in 0.1 M PBS/150 mM NaCl, pH 7.4; detection potential: 0.3 V; measurement time: 10 s.

..

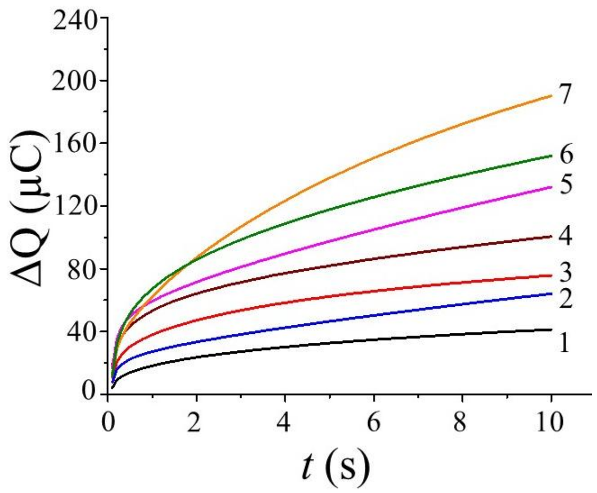

Figure S2. Representative CC responses to different concentrations of total uPA ranging from 1.0 aM to 1.0 pM in human serum: (1) 1.0 aM (black), (2) 10 aM (blue), (3) 0.1 fM (red), (4) 1.0 fM (wine), (5) 10 fM (magenta), (6) 100 fM (olive green), (7) 1.0 pM (orange). Responses were recorded in 0.1 M PBS/150 mM NaCl, pH 7.4; detection potential: 0.3 V; measurement time: 10 s.

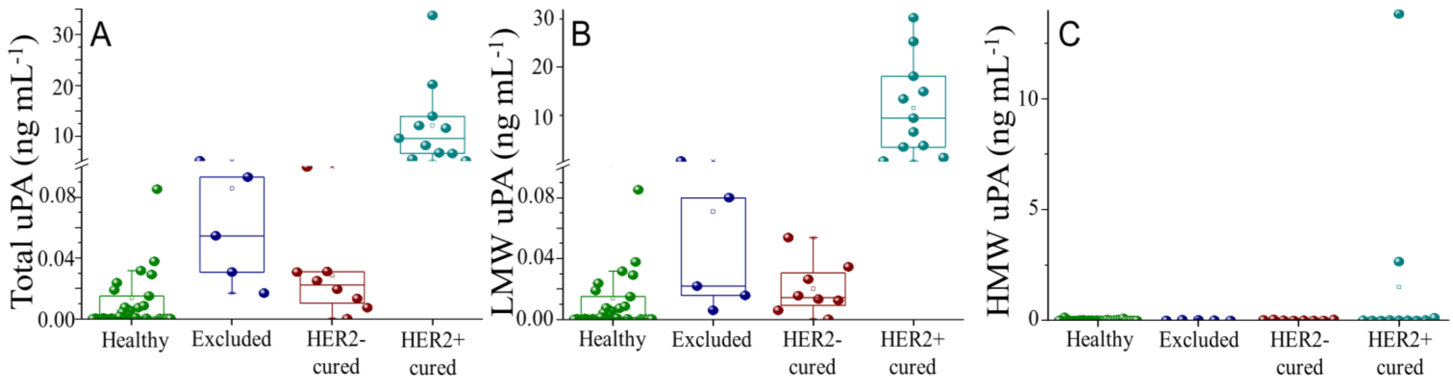

Figure S3. Correlation between serum (**A**) total uPA, (**B**) LMW uPA, and (**C**) HMW uPA and patients' *HER2* status based on analysis of serum samples collected from healthy volunteers (n = 30), excluded from formal analysis (n = 5), and cured *HER2*-negative patients (n = 8) and allegedly *HER2*-positive cancers (HER-2/*neu* overexpressed and borderline-expressed) patients (n =11). Serum samples were diluted to 10% serum before being examined using CC at 0.3 V and 10 s, with 0.1 M PBS/150 mM NaCl, pH 7.4. In all plots, the middle line indicates the median, the error bar means the upper and lower values (according to the 1.5 × IQR criterion, extending to the most extreme non-outlier values), the box refers to the range in which the middle 50% of all data points are, and the upper and lower boxes mean the upper and lower quartiles, which are 25% of the data more or less than those values. Statistical analysis was carried out using Origin 8.5.

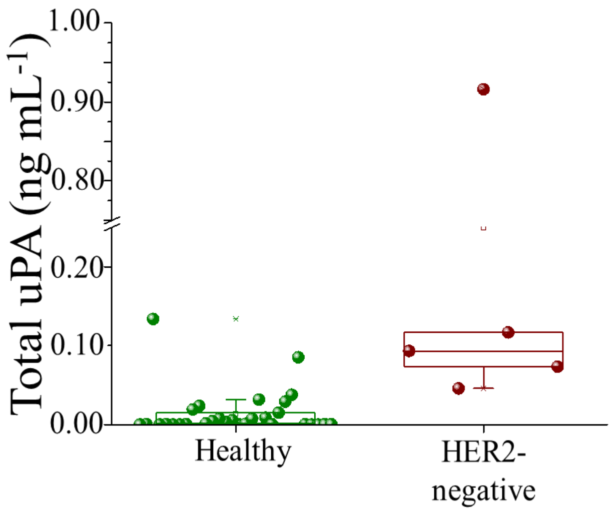

Figure S4. Correlation between uPA levels in patients with HER2-negative gastric and cardia cancers excluded from formal analysis (n = 6, wine) and healthy individuals' group (n = 30, green).

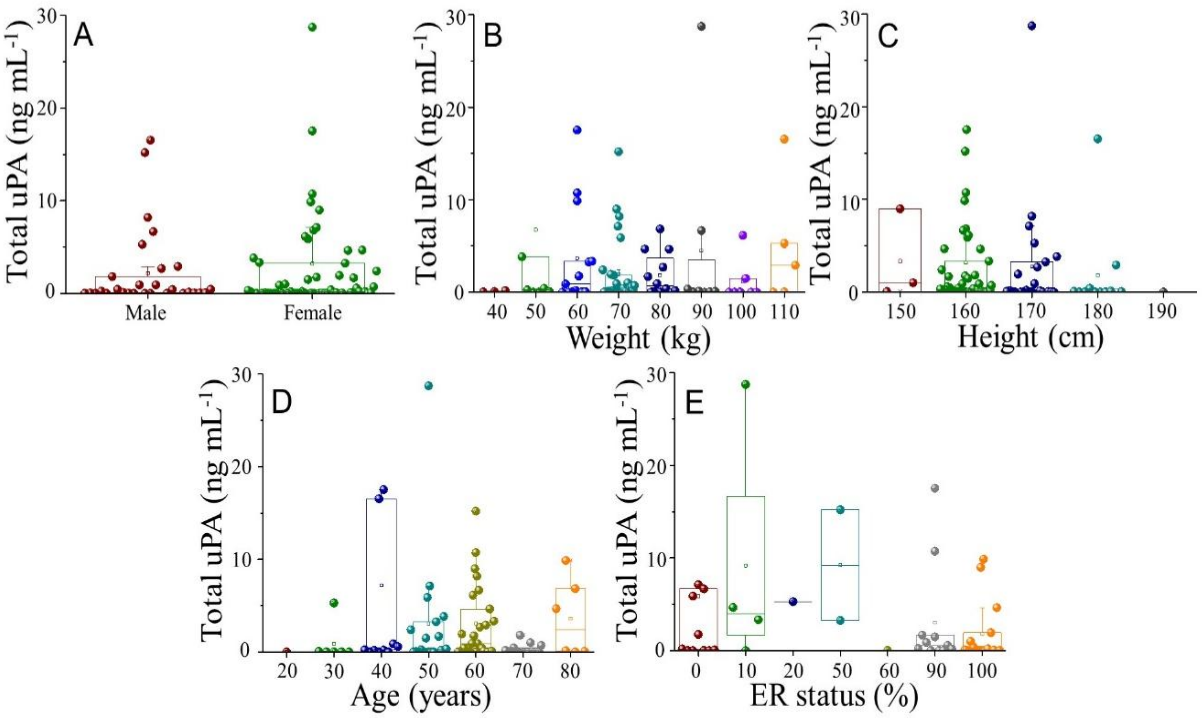

Figure S5. Total uPA values obtained from the MBs-aptamer-aptamer assay were plotted against various clinical and physical parameters: (A) gender (B) weight, (C) height, (D) age, (E) ER status. 85 serum samples were diluted to 10% serum before being examined using CC at 0.3 V and 10 s, with 0.1 M PBS/150 mM NaCl, pH 7.4. In all plots, the middle line indicates the median, the error bar means the upper and lower values (according to the 1.5 × IQR criterion, extending to the most extreme non-outlier values), the box refers to the range in which the middle 50% of all data points are, and the upper and lower boxes mean the upper and lower quartiles, which are 25% of the data more or less than those values. Statistical analysis was carried out using Origin 8.5.

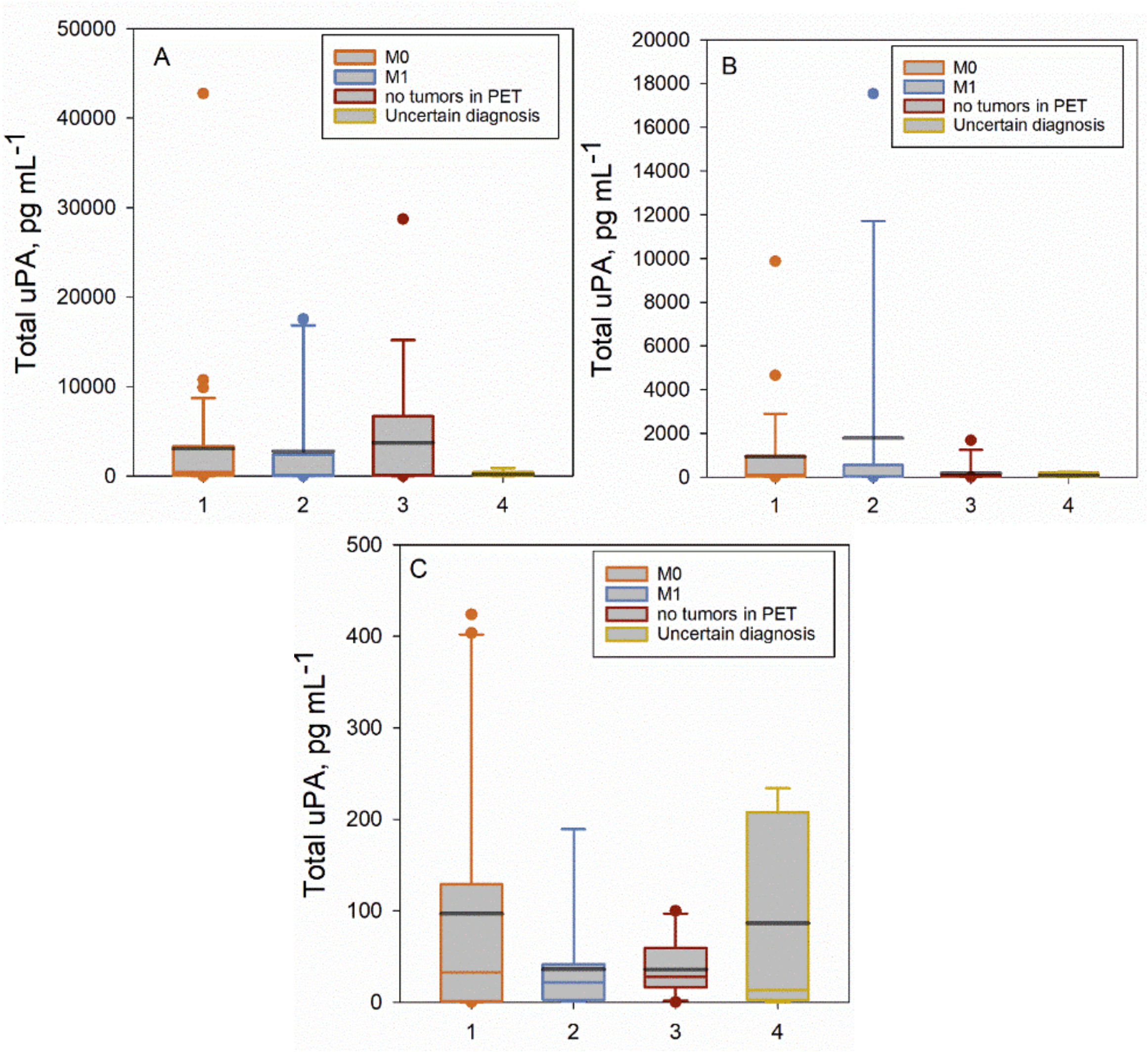

Figure S6. Box plots representing serum total uPA levels in four groups of 77 cancer patients (1: with no metastasis; 2: with metastasis; 3: with no tumor found at the time of liquid biopsy collection (allegedly cured patients); and 4: with tumor status unspecified in patient's e-journal); horizontal black lines are median values, and perecntiles are represented by horizontal boxes with error bares (Sigma Plot 13). (**A**) data for all patients, (**B**) data with *HER2+* cancers excluded; and (**C**) data with *HER2+* cancers and cancers with a borderline expression of HER-2/*neu* excluded. *HER2+* cancers include two borderline expression cases for which gene amplification analysis confirmed *HER2+* diagnosis. The median values for 1 – 4 are: (A) 3171, 2800, 3828, 285; (B) 971, 1811, 230, 102; and (C) 97, 37, 38, and 88 pg mL^{-1}, correspondingly.

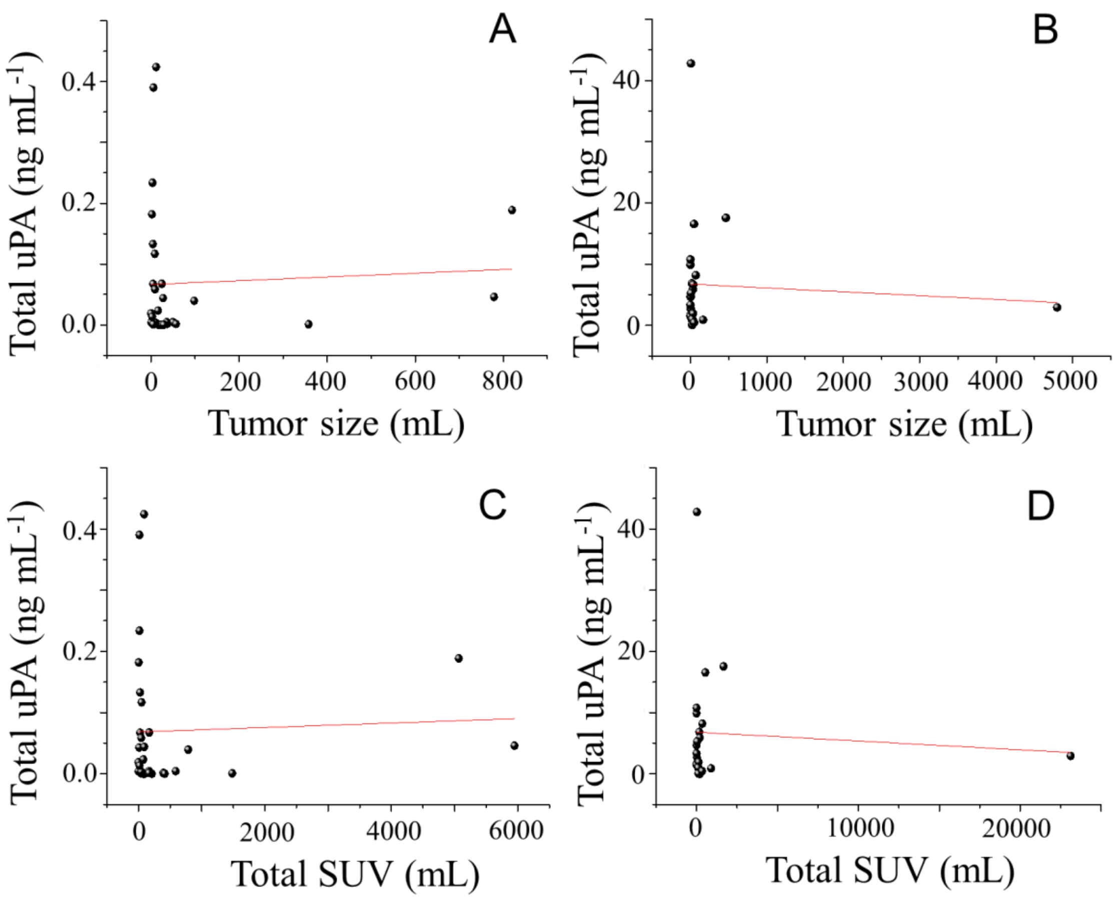

Figure S7. Correlations between serum total uPA and (**A, B**) the tumor size and (**C, D**) total standardized uptake value (total SUV) in (**A, C**) HER2 negative and (**B, D**) HER2 positive cancers and cancers with a borderline expression of HER-2/*neu*. (**A**): r^2 = 0.057; (**B**): r^2 = -0.067; (**C**): r^2 = 0.045; (**D**): r^2 = -0.074. The total SUV represents total metabolic activity of the whole tumor.

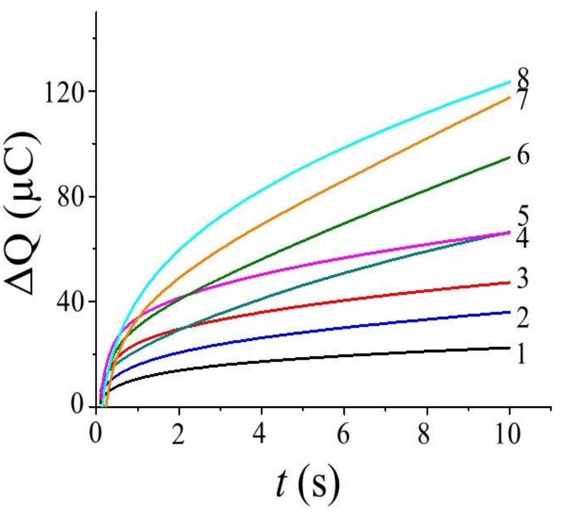

Figure S8. Representative CC responses to different concentrations of HMW uPA ranging from 1.0 aM to 10 pM in human serum: (1) 1.0 aM (black), (2) 10 aM (blue), (3) 0.1 fM (red), (4) 1.0 fM (dark cyan), (5) 10 fM (magenta), (6) 100 fM (olive green), (7) 1.0 pM (orange), (8) 10 pM (cyan). Responses were recorded in 0.1 M PBS/150 mM NaCl, pH 7.4; detection potential: 0.3 V; measurement time: 10 s.

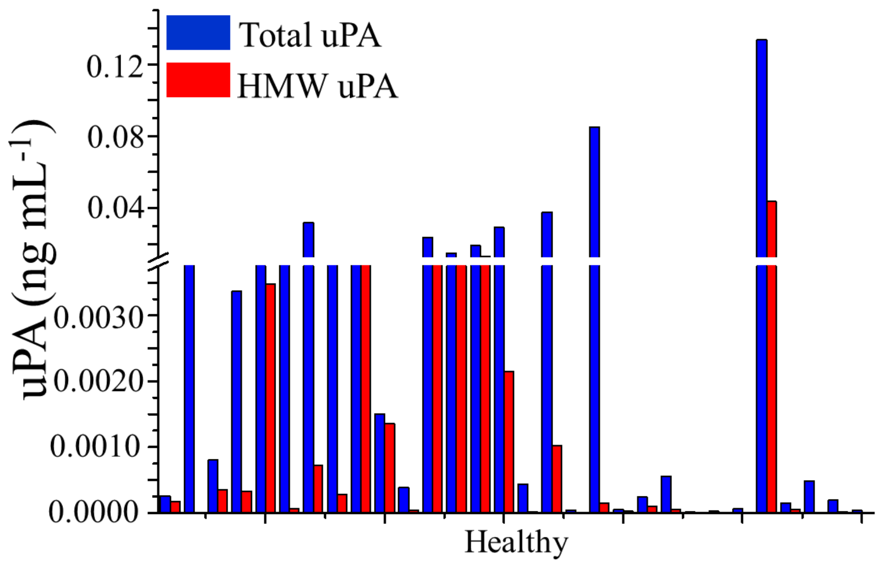

Figure S9. Serum total uPA (blue) and HMW uPA (red) levels in healthy individuals (n = 30).

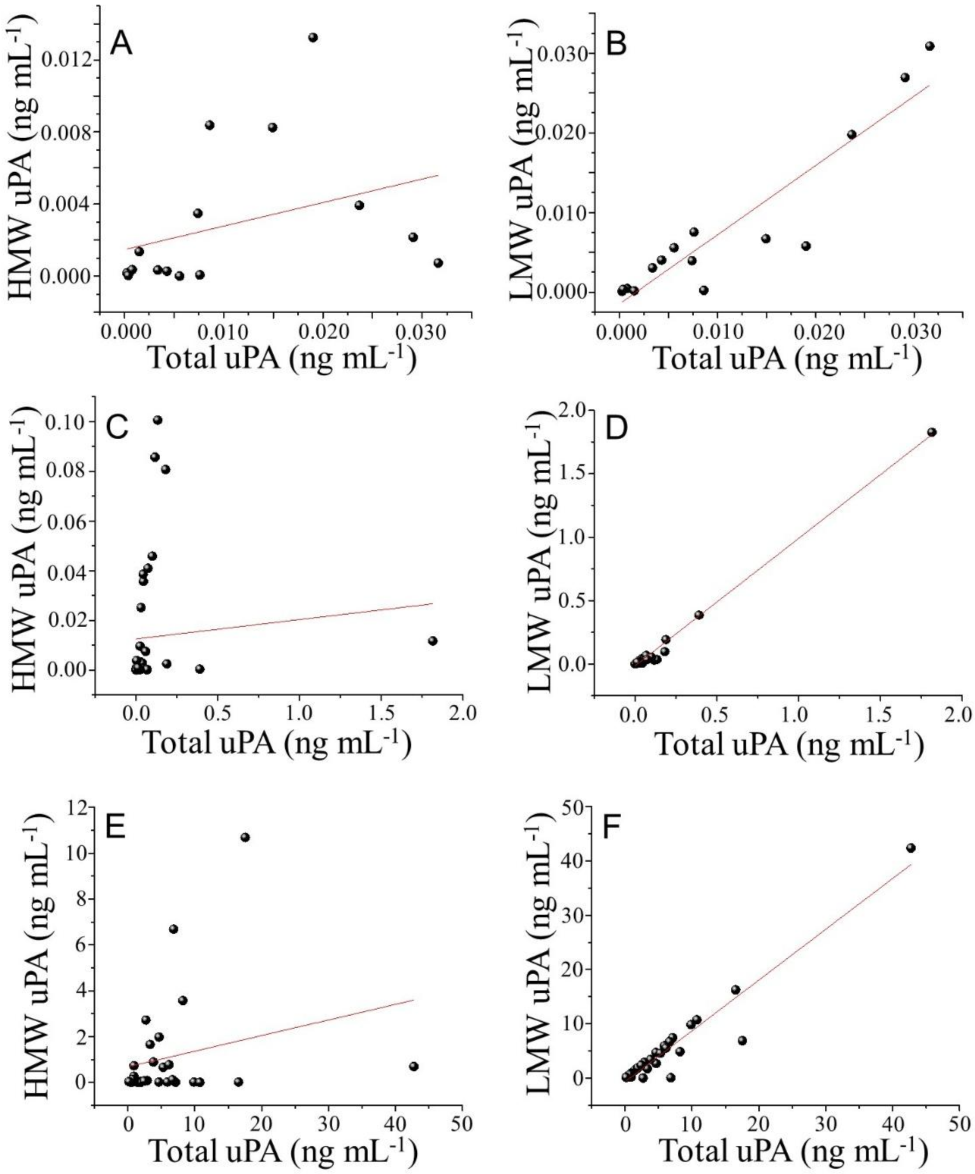

Figure S10. Correlations between concentrations of serum total uPA and either serum HMW uPA (**A, C, E**) or serum LMW uPA (**B, D, E**) in (**A, B**) a healthy cohort, and (**C, D**) *HER2*-negative and (**E, F**) *HER2* positive/HER-2/*neu* borderline-expressed cancer patients. (**A**): r^2 = 0.344; (**B**): r^2 = 0.925; (**C**): r^2 = 0.089; (**D**): r^2 = 0.996; (**E**): r^2 = 0.242; (**F**): r^2 = 0.960.

References:

1 Díaz-Fernández, A., Ferapontov, A., Vendelbo, M. H. & Ferapontova, E. E. Electrochemical Cellulase-Linked ELASA for Rapid Liquid Biopsy Testing of Serum HER-2/neu. *ACS Measur. Sci. Au* **3**, 226-235 (2023).

2 Fapyane, D. & Ferapontova, E. E. Electrochemical assay for a total cellulase activity with improved sensitivity. *Anal. Chem.* **89**, 3959-3965 (2017).
