# Supplementary material for: Differentiating borderline HER2-expressing and HER2-positive cancers from other subtypes using serum urokinase plasminogen activator
Source: Br J Cancer. 2026 May 20;135(4):649–58. doi: 10.1038/s41416-026-03471-5 (PMC13427726; doi:10.1038/s41416-026-03471-5)
Supplement: Supplementary file 2 — Supporting Information: Table S1 [file 41416_2026_3471_MOESM2_ESM.pdf]

Table S1. Patients' demographic, cancer biopsy results and other relevant clinical data

Patient	Sex (	Age	Height	Weight	Cancer type	Subtype	HER2 expression	HER2 gene	HER2 score	TNM stage	Size (ml)	Total (SUVxml)
1	1	63	166	55	Breast	Invasive ductal carcinoma	Normal		1	T1N1M0	0,7	2,5
2	1	57	176	110	Breast	Invasive lobular carcinoma	Negativ		0	T2N0M1	15,9	81,8
3	1	69	160	61	Breast	Invasive ductal carcinoma	Borderline	Normal	2	T2N0M0	6,09	30
4	1	54	160	102	Breast	Invasive ductal carcinoma	Borderline	Normal	2	T1N0M0	0,14	0,5
5	1	46	168	68	Breast	Invasive ductal carcinoma	Borderline	Normal	2	T0N0M1	465	1678
6	1	47	170	75	Breast	Adenocarcinoma	Normal		1	T4N0Mx	17,6	77,9
7	1	31	165	78	Breast	Invasive ductal carcinoma	Low		1	0		
8	2	73	160	79	Esophagus	Mucinous adenocarcinoma	Normal		0	T4aN0M0	23,4	89,3
9	2	84	180	80	Esophagus	Squamous cell carcinoma	Normal		0	cT3N1Mx	50,1	591
10	2	63	179	83	Gastric	Adenocarcinoma	Borderline	Normal	2	T3N1M0	29,6	207
11	2	69	165	71	Esophagus	Poorly differentiated adenocarcinoma	Overexpression		3	cT3N2Mx	17,5	143
12	1	75	165	75	Breast	Invasive lobular carcinoma	Normal		1	0		
13	1	42	170	62	Breast	Invasive ductal carcinoma	?	Normal	2	T2N1M0	3,53	13,4
14	1	35	168	61	Breast	Invasive ductal carcinoma	Normal		1	T1N0M0	0,28	0,8
15	1	83	165	80	Esophagus	Adenocarcinoma	Overexpression		3	T3N3M0	21,5	175
16	2	67	175	82	Esophagus	Poorly differentiated adenocarcinoma	Borderline	Normal	2	T3N1M0	7,03	36,1
17	1	27	172	110	Breast	Carcinoma	Normal		1	T2NxM0 ?	14,6	122
18	2	62	178	77,3	Esophagus	Adenocarcinoma	Overexpression		3	T3N1M0	71,5	367
19	2	51	168	75	Esophagus	Earlier squamous cell carcinoma	Normal			0		
20	1	67	182	101	Breast	Earlier invasive ductal carcinoma. Actually no sickness	Normal		0	0		
21	2	45	195	90	Esophagus	Adenocarcinoma	Normal		1	T3N0M0	6,36	36,8
22	2	59	186	82	Esophagus	Poorly differentiated squamous cell carcinoma	Normal		1	T3N2M1	98,3	788
23	1	76	165	58	Breast	Carcinoma	Normal		1	T2N3M1	10,2	43,3
24	2	64	187	75	Esophagus	Adenocarcinoma	?		2	T4N0M0	12,1	56,8
25	1	68	165	89	Breast	Carcinoma	Normal		1	T3N0M0	5,98	18,8
26	2	60	180	95	Gastric	Adenocarcinoma	Normal		1	T3N1M0	8,83	50
27	1	67	180	100	Breast	Carcinoma	Normal		0	T3N0M0	36,6	393
28	2	65	183	140	Esophagus	Poorly differentiated adenocarcinoma	Overexpression		3	T3N3M1	4801	23118
29	2	63	166	53,4	Esophagus	Squamous cell carcinoma	Normal		0	T4N0M0	12	89,2
30	2	61	181	104	Esophagus	Poorly differentiated adenocarcinoma	Normal		0	T3N1M0	25,5	215
31	2	77	168	72	Esophagus	Adenocarcinoma	Overexpression		3	0		
32	1	36	162	62	Breast	Invasive ductal carcinoma	Normal		1	0		
33	1	80	162	66	Breast	Invasive ductal carcinoma. Dominant intraductal component	Borderline	Normal	2	T1N1M0	0,88	5,4
34	2	88	178	77	Esophagus	Adenocarcinoma	Normal		1	T3N0M0	4,36	26,9
35	1	44	167	71	Breast	Carcinoma	Normal		1	T3N3M1	820	5067
36	1	37	168	74	Breast	Invasive ductal carcinoma	Normal		0	T3N0M0	4,92	25,7
37	1	43	162	55	Breast	Carcinoma	Overexpression		3	T2N1M0	7,46	34,1
38	2	52	171	104	Esophagus	Squamous cell carcinoma	Normal		1	T2N0M0		
39	1	77	175	107	Breast	Invasive ductal carcinoma	Normal		1	T0N3M1	35,8	167
40	1	64	172	78	Breast	Carcinoma	Borderline	Normal	2	T3N1M0	33,7	134
41	2	71	185	93	Gastric	Undifferentiated pleomorphic sarcoma	Normal		0	T4N0M0	779	5950
42	1	73	159	75	Breast	Invasive ductal carcinoma	Borderline	Normal	2	T2N0M0	21,1	97,6
43	1	79	152	69	Breast	Carcinoma	Borderline	Normal	2	T0N0M1	22,6	113
44	2	55	165	55	Esophagus	Earlier poorly differentiated adenocarcinoma. Actually no sickr	Normal		1	n.a.	3,47	12,7
45	1	39	168	65	Breast	Carcinoma	Normal		1	T4N0M0	4,78	17,3

Patient	Sex (	Age	Height	Weight	Cancer type	Subtype	HER2 expression	HER2 gene	HER2 score	TNM stage	Size (ml)	Total (SUVxml)
46	1	53	175	68	Breast	Carcinoma	Overexpression		3	0		
47	1	46	168	63	Breast	Invasive ductal carcinoma	Borderline	Normal	2	T0N3M1	167	907
48	2	73	160	75,3	Esophagus	Mucinous adenocarcinoma	Normal		0	T4N0M0 ?	9,25	45,9
49	1	69	169	62	Breast	Invasive ductal carcinoma	Borderline	Gene amplific	2	T0N3M0	1,82	8,3
50	2	59	177	88	Esophagus	Poorly differentiated adenocarcinoma	Normal		1	T3N3M1	358	1483
51	1	69	163	80	Breast	Invasive ductal carcinoma	Borderline	Normal	2	T1N0M0	0,01	0
52	2	80	180	73	Esophagus	Adenocarcinoma	Normal		1	T3N2M0	24,7	171
53	1	57	172	74	Breast	Carcinoma	Overexpression		3	0		
54	1	42	169	90	Breast	Earlier invasive ductal carcinoma. Actually no sickness	Normal		1	0		
55	2	60	170	55	Esophagus	Adenocarcinoma	Normal		1	T3N1M1	27,8	91,9
56	1	51	163	72	Breast	Invasive ductal carcinoma	Borderline	Normal	2	T0N0M1	33,5	212
57	1	68	151	78	Breast	Invasive ductal carcinoma	Overexpression		3	0		
58	1	65	164	73	Breast	Earlier invasive ductal carcinoma. Actually no sickness	Normal		1	0		
59	2	78	167	75	Esophagus	Adenocarcinoma	Borderline		1	T3N3M1	46,6	347
60	1	74	168	74	Breast	Invasive lobular carcinoma	Normal		1	T4N0M1	56,8	164
61	1	57	169	87	Breast	Earlier invasive ductal carcinoma. Actually no sickness	Borderline	Normal	2	0		
62	1	61	165	82	Breast	Carcinoma	Normal		0	T2N1M0	28,9	416
63	1	42	168	76	Breast	Earlier invasive ductal carcinoma. Actually no sickness	Borderline	Gene amplific	2	0		
64	1	58	163	77,5	Esophagus	Nothing on scan day, earlier planocellulært karcinom	?	?	0-1?	0		
65	1	55	177	94	Breast	Earlier invasive ductal carcinoma. Actually no sickness	Overexpression		3	0		
66	1	42	166	73,5	Breast	Earlier invasive ductal carcinoma. Actually no sickness	Borderline	Normal	2	0		
67	1	77	160	43	Gastric	Poorly differentiated adenocarcinoma	Normal		1	0		
68	2	76	176	75	Esophagus	Intestinal metaplasia	?	?	?	0		
69	1	81	166	86	Breast	Invasive ductal carcinoma	Overexpression		3	T0N3M0	2,37	12,7
70	1	77	172	47	Breast	Carcinoma	Normal		0	T4N3M1	16,1	78
71	1	57	173	85	Breast	Invasive ductal carcinoma	Normal		1	n.a.	2,43	6,8
72	1	65	167	61	Breast	Carcinoma	Overexpression		3	T2N0M0	1,35	4
73	1	76	160	41	Esophagus	Signet ring cell carcinoma	Normal		0	T4N0Mx	2,85	17
74	2	47	180	125	Cardia	Adenocarcinoma	Overexpression		3	T3N2M1	46,9	551
75	1	66	168	73	Breast	Invasive ductal carcinoma	Overexpression		3	0		
76	1	39	170	107	Breast	Carcinoma	Overexpression		3	T4N1M0	8,05	45,8
77	1	66	162	92	Breast	Invasive ductal carcinoma	Overexpression		3	0		
78	1	52	176	67	Breast	Carcinoma	Ultra low expression			T2N3M0	35,5	302
79	1	71	170	95	Gastric	No malignancies found	Intet svar			n.a.	n.a.	n.a.
80	1	57	170	58	Breast	Papillary ductal carcinoma	Overexpression		3	n.a.	n.a.	n.a.
81	1	75	161	74	Breast	Carcinoma	Borderline	Normal	2	n.a.	n.a.	n.a.
82	1	60	169,5	100	Breast	Invasive ductal carcinoma	Overexpression		3	n.a.	n.a.	n.a.
83	1	73	160	46	Breast	Invasive ductal carcinoma	Borderline	Normal	2	n.a.	n.a.	n.a.
84	1	54	163	71	Breast	Invasive ductal carcinoma	Overexpression		3	n.a.	n.a.	n.a.
85	1	57	169	95	Breast	Lobular carcinoma	Normal		1	n.a.	n.a.	n.a.
86	1	61	175	80	Breast	Invasive ductal carcinoma	Normal		1	n.a.	n.a.	n.a.
87	2	55	186	68	Esophagus	Squamous cell carcinoma	Normal		0	n.a.	n.a.	n.a.
88	1	52	171	68	Breast	Invasive ductal carcinoma	Overexpression		3	n.a.	n.a.	n.a.
89	1	36	164,5	63	Breast	Mucinous adenocarcinoma	Borderline	Normal	2	n.a.	n.a.	n.a.
90	1	65	170	105	Breast	Invasive ductal carcinoma	Normal		1	n.a.	n.a.	n.a.

Patient	Sex (	Age	Height	Weight	Cancer type	Subtype	HER2 expression	HER2 gene	HER2 score	TNM stage	Size (ml)	Total (SUVxml)
91	1	76	175	58	Breast	Ductal carcinoma	Normal		1	n.a.	n.a.	n.a.
92	1	33	170	80	Breast	Invasive ductal carcinoma	Normal		1	n.a.	n.a.	n.a.
93	1	58	162	100	Breast	Ductal carcinoma	Normal		0	n.a.	n.a.	n.a.
94	1	33	171	56	Breast	Invasive ductal carcinoma	Overexpression		3	n.a.	n.a.	n.a.
95	2	72	178	78	Esophagus	Adenocarcinoma	Borderline	Normal		n.a.	n.a.	n.a.
96	2	52	178	77	Esophagus	Adenocarcinoma?	Normal			n.a.	n.a.	n.a.
97	1	72	160	48	Esophagus	Poorly differentiated carcinoma	Normal		1	n.a.	n.a.	n.a.
98	1	29	164	85	Breast	Invasive ductal carcinoma	Borderline	Normal	2	n.a.	n.a.	n.a.
99	2	66	166	60,5	Esophagus	Squamous cell carcinoma	Normal			n.a.	n.a.	n.a.
100	1	76	164	53	Breast	Invasive ductal carcinoma	Borderline	Normal	2	n.a.	n.a.	n.a.

Sex: 1 – female; 2 – male; Height: in cm; Weight: in kg; Marked with "?" are undefined coditions; in TNM stage: "0" - PET scanning showed no detectable tumors to the moment of serum samples collection; n.a.: not available data.
